# Production of Antimicrobial and Antioxidant Metabolites by *Penicillium crustosum* Using Lemon Peel as a Co-Substrate in Submerged Fermentation

**DOI:** 10.3390/foods15020348

**Published:** 2026-01-18

**Authors:** Arely Núñez-Serrano, Refugio B. García-Reyes, Juan A. Ascasio-Valdés, Cristóbal N. Aguilar-González, Alcione García-González

**Affiliations:** 1 Facultad de Ciencias Químicas, Universidad Autónoma de Nuevo León (UANL), Av. Universidad S/N, Cd. Universitaria, San Nicolás de los Garza 66455, Nuevo León, Mexico; arely.nunezsrr@uanl.edu.mx (A.N.-S.); refugio.garciary@uanl.edu.mx (R.B.G.-R.); 2 Bioprocesses and Bioproducts Research Group, Food Research Department, School of Chemistry, Universidad Autónoma de Coahuila, Unidad Saltillo, Saltillo 25280, Coahuila, Mexico; alberto_ascaciovaldes@uadec.edu.mx (J.A.A.-V.); cristobal.aguilar@uadec.edu.mx (C.N.A.-G.)

**Keywords:** antimicrobial, antioxidants, *Penicillium crustosum*, lemon peel

## Abstract

Fungal secondary metabolites are valuable sources of natural antioxidants and antimicrobials. This study evaluated the submerged fermentation of *Penicillium crustosum* OR889307 supplemented with lemon peel as a co-substrate to enhance the production of bioactive compounds. Lemon peel was selected for its phenolic precursors and sustainable availability as an agro-industrial byproduct. Crude extracts, aqueous and organic fractions, and molecular-weight partitions were assessed for antioxidant activity using the DPPH assay and for antimicrobial activity against *Escherichia coli*, *Staphylococcus aureus*, methicillin-resistant *S. aureus* (MRSA), *Pseudomonas aeruginosa*, and *Candida albicans*. Semi-purified extracts from co-substrate fermentations exhibited enhanced bioactivity, showing MIC values of 185 µg/mL against *P. aeruginosa* and 225 µg/mL against MRSA, along with strong ABTS radical-scavenging capacity (238.95 ± 2.17 µmol TE). RP-HPLC-ESI-MS profiling revealed phenolic acids, flavanones, flavonols, and lignans, including ferulic acid 4-O-glucoside, bisdemethoxycurcumin, secoisolariciresinol, and quercetin 3-O-xylosyl-glucuronide. These findings demonstrate that lemon peel supplementation promotes the biosynthesis of antimicrobial and antioxidant metabolites by *P. crustosum*. This approach supports sustainable agro-waste valorization and offers a promising strategy for obtaining natural bioactive compounds with potential applications in food preservation and health-related formulations.

## 1. Introduction

Antibiotic resistance represents a critical global health challenge, threatening the effectiveness of antibiotics in treating bacterial infections and increasing both the severity and incidence of infectious diseases. Relevant pathogens such as *Escherichia coli*, *Staphylococcus aureus*, methicillin-resistant *Staphylococcus aureus* (MRSA), *Pseudomonas aeruginosa*, and *Candida albicans* have become increasingly difficult to treat due to their growing resistance profiles. The overuse of antimicrobials in human and veterinary medicine, as well as in livestock farming and agricultural practices, is a major driver of bacterial resistance [1]. Therefore, exploring and developing alternative strategies and novel sources of antimicrobial compounds are essential.

Natural products derived from fungal secondary metabolism have gained increasing attention due to their diverse bioactivities. These microorganisms have evolved complex metabolic pathways that produce a wide range of secondary metabolites, including antimicrobial, antifungal, antioxidant, and antiproliferative agents [2]. The discovery and exploitation of fungal-derived compounds have significantly contributed to pharmaceutical development. For example, the use of penicillin, an antibiotic produced by *Penicillium notatum*, revolutionized modern medicine [3]. Moreover, several other compounds have been reported from various filamentous fungi. For instance, penixylarins produced by *Penicillium crustosum* PRB-2 and *Xylaria* sp. HDN13-249 exhibits antibacterial activity against *Mycobacterium phlei*, *Bacillus subtilis*, and *Vibrio parahaemolyticus* [4]. Similarly, ketone-related compounds isolated from solid-state fermentation of *P. crustosum* using ethyl acetate have demonstrated antimicrobial activity against *Micrococcus luteus*, *S. aureus*, and *C. albicans* [5]. Ethyl acetate extracts from endophytic strains such as *Penicillium roqueforti* (CGF-1) and *Trichoderma reesei* (CGF-11) have also shown antimicrobial activity against *Pseudomonas syringae*, with positive results attributed to the presence of bioactive compounds including ferulic acid, cinnamic acid, and quercetin [6], which are also known for their antioxidant potential [7]. Other fungal strains, such as *Aspergillus* spp., have been reported to produce phenolic and flavonoid compounds. For example, *Aspergillus terreus* AUMC 15444 and *A. nidulans* AUMC 1544 produce gallic acid, cinnamic acid, *p*-coumaric acid, and ferulic acid, all of which exhibit antimicrobial activity against *S. aureus* and *Candida* spp. [8].

Submerged fermentation is commonly used to produce fungal metabolites of interest. This approach enables precise control of nutrient supply, pH, temperature, mixing, and aeration, all of which influence fungal metabolic pathways [9,10]. However, to enhance the yield and diversity of secondary metabolites, the addition of co-substrates is often required, as they may function as metabolic enhancers or precursor molecules.

Among agro-industrial residues, citrus by-products such as lemon peel represent a sustainable and nutrient-rich alternative for fungal fermentation. Lemon peel contains carbohydrates, flavonoids, phenolic acids, and pectic substances that can stimulate fungal growth and secondary metabolism. In addition, it provides precursors for phenolic and flavonoid biosynthesis, including *p*-coumaric and ferulic acids, which may serve as co-metabolic substrates in bioprocesses [11].

The valorization of citrus by-products contributes to waste reduction and promotes the development of functional ingredients derived from microbial biotransformation. Despite this potential, the specific influence of lemon peel on the production of extracellular bioactive metabolites by *Penicillium* species remains insufficiently explored. Therefore, this study aimed to evaluate the effect of lemon peel as a co-substrate in the submerged fermentation of *Penicillium crustosum* OR889307 to produce antioxidant and antimicrobial metabolites. This work provides the first evidence that lemon peel co-substrate enhances the extracellular biosynthesis of phenolic and flavonoid metabolites by *P. crustosum*.

## 2. Materials and Methods

### 2.1. Reagents and Strains

All chemicals used in this study were of analytical grade. Potato dextrose agar (PDA) from BD Bioxon, (Ciudad de Mexico, Mexico), potato dextrose broth (PDB) and Müller-Hilton broth from BD Bioxon, (Le Pont de Claix, France), ethyl acetate and ethanol from PQM Fermont, (Monterrey, Mexico), citric pectin, Trolox, DPPH, ABTS, Luria–Bertani (LB) agar, and Amberlite^TM^ XAD16 resin were purchased from Sigma-Aldrich (St. Louis, MO, USA).

Pathogenic bacterial strains, including *Escherichia coli* ATCC 11229, *Staphylococcus aureus* ATCC 6538, and *Pseudomonas aeruginosa* ATCC 27853, were obtained from the Graduate Department of the School of Chemical Sciences, UANL, Mexico, and methicillin-resistant *Staphylococcus aureus* (MRSA) was provided by the Clinical Pathology Department of the Hospital Universitario Dr. José Eleuterio González, Universidad Autónoma de Nuevo León, Mexico. The *P. crustosum* strain OR889307 and *Candida albicans* ATCC 18804 were provided by the Laboratory of Electrochemistry and Environmental Biocatalysis of the School of Chemical Sciences, UANL.

Lemon peels (LP) were collected from a local juice shop in Monterrey, Nuevo León, Mexico.

### 2.2. Fungal Strain and Growth Conditions

The strain *Penicillium crustosum* OR889307 was previously isolated in Monterrey, Nuevo León, Mexico. The strain was cultivated on potato dextrose agar (PDA) supplemented with 2% (*w*/*v*) citric pectin at 35 °C for 5 days, as described in our previous reports [12].

### 2.3. Cultivation Conditions for the Production of Antimicrobial and Antioxidant Compounds

The effect of time and the addition of a co-substrate on the production of antimicrobial and antioxidant compounds by *Penicillium crustosum* OR889307 was evaluated under submerged fermentation conditions. Lemon peel, previously dried, pulverized, and sieved, was used as a co-substrate.

The strain was inoculated at a concentration of 1 × 10^6^ spores/mL into a 250 mL Erlenmeyer flask containing 100 mL of potato dextrose broth. This culture, without any additional co-substrate, was referred to as the “PDB” sample. A second treatment was prepared using the same conditions, incorporated as a co-substrate at 2% (*w*/*v*), based on previous optimization assays [12]. This culture was referred to as the “PDB+LP” sample.

Fermentations were carried out for 7 days at 45 °C, under constant orbital shaking at 120 rpm in triplicate. Both media were supplemented with 2.5 g/L of MnSO_4_ and adjusted to a pH of 5.0. These conditions were selected based on previously optimized conditions that resulted in maximal enzyme production by *P. crustosum* OR889307 under similar submerged fermentation settings [13]. Every 24 h, a representative sample of the supernatant was collected and filtered through Whatman No. 1 filter paper, followed by a 0.45 µm cellulose membrane; this filtrate was defined as “crude sample”. The crude sample obtained at the fermentation time point exhibiting the highest antimicrobial activity was selected for subsequent extraction and purification steps.

### 2.4. Extraction of Antimicrobial Compounds

Bioactive compounds were extracted from crude samples (PDB and PDB+LP) using ethyl acetate (three successive extractions to exhaustion) in a separatory funnel. The organic (ethyl acetate) layers were pooled and concentrated under reduced pressure at 50 °C. Two fractions were obtained: the ethyl acetate fraction (“organic phase”) and the remaining aqueous fraction (“aqueous phase”). Both were evaluated for antimicrobial activity.

### 2.5. Fractionation and Evaluation of Fermented Extracts

Crude samples were fractionated using a 10 kDa cut-off membrane. Samples were placed in 15 mL Amicon filters and centrifuged at 4500 rpm for 20 min at 4 °C, yielding fractions containing >10 kDa and <10 kDa molecules. Both fractions were evaluated for antimicrobial activity to determine the most active molecular range.

### 2.6. Semi-Purification of Crude Samples Using Column Chromatography

Semi-purification was carried out by affinity chromatography using Amberlite XAD16 resin. Columns were first eluted with distilled water to remove impurities, followed by elution with absolute ethanol to recover the target organic fraction. The solvent was evaporated to obtain a dry powder, which was dissolved in distilled water and filtered through 0.45 µm membranes [14]. The resulting solutions were designated as semi-purified samples.

### 2.7. Antimicrobial Activity Measurement

Antimicrobial activity was first assessed qualitatively using the agar disk diffusion method. *Escherichia coli* (Gram-negative), *Staphylococcus aureus* (Gram-positive), and *Candida albicans* were used as test organisms. Bacterial suspensions were adjusted to 0.5 McFarland. All tests were conducted in triplicate (*n* = 3), and inhibition zones were measured with a digital caliper.

Microbial cultures were grown on Luria–Bertani (LB) agar, diluted to 10^8^ CFU/mL, and spread onto LB plates. Whatman No. 1 paper disks (6 mm) were loaded with 20 µL of crude sample. Sterile water (20 µL) served as the negative control. Positive controls included streptomycin (2 mg/mL, 20 µL) for *E. coli* and *S. aureus*, and fluconazole (2 mg/mL, 20 µL) for *C. albicans*. Crude extracts from uninoculated PDB and PDB+LP media were included as media controls. Plates were incubated at 37 °C for 24 h, and inhibition zone diameters were recorded [8].

For quantitative assessment, the microdilution method was used. First, fermentation time points at which crude samples exhibited maximal inhibition were identified. These samples were then compared with their corresponding extracted, fractionated, and semi-purified forms. Pathogen cultures were prepared as described above. Each well contained 100 µL microbial suspension and 100 µL Müller–Hinton broth. Controls included sterile water (negative control) and the same antibiotics used in qualitative testing. After adding samples (100 µL), plates were incubated for 24 h at 37 °C. Antimicrobial activity was calculated as percentage inhibition based on turbidity changes at 660 nm [15], in 96 well plates using Multiskan^TM^ SkyHigh Microplate Spectrophotometer (Thermoscientific, Life Technologies Holdings Pte Ltd., Singapore).

### 2.8. Minimal Inhibitory Concentration (MIC) and the Half-Maximal Inhibitory Concentration (IC_50_) Values

MIC was determined for the sample with the highest inhibitory activity (semi-purified extracts). Samples were rehydrated in sterile distilled water to obtain concentrations from 500 to 0.6 µg/mL. MIC assays followed the microdilution method described above and were performed against *E. coli*, *S. aureus*, MRSA, and *P. aeruginosa*. Bacterial suspensions were adjusted to 0.5 McFarland. Plates were incubated for 24 h at 37 °C, and MIC was defined as the lowest concentration that completely inhibited visible growth, confirmed by lack of turbidity at 660 nm [15] using Microplate Spectrophotometer.

*IC_50_* values were determined by nonlinear regression in Origin Pro 2023 (OriginLab Corporation, Northampton, MA, USA, v9.80). Inhibition data were log-transformed (log_10_ concentration, µg/mL) and fitted to a sigmoidal dose–response curve using Equation (1):

(1) $$y=A_{2}+\frac{A_{1}-A_{2}}{1+{\left(\frac{x}{{IC}_{50}}\right)}^{p}}$$

### 2.9. Analysis of Antimicrobial Compounds Using RP-HPLC-ESI-MS

Bioactive compounds were identified by reverse-phase high-performance liquid chromatography (RP-HPLC) coupled with electrospray ionization mass spectrometry (ESI-MS). Analyses were performed on HPLC system, (ProStar 410, Palo Alto, CA, USA) equipped with an autosampler, ternary pump, and photodiode array (PDA) detector (ProStar 330, Palo Alto, CA, USA). A Varian 500-MS ion trap mass spectrometer (Palo Alto, CA, USA) with an ESI source was used. A 10 µL injection was loaded onto a Denali C18 column (150 × 2.1 mm, 3 µm).

The mobile phase consisted of methanol (washing), formic acid (0.2% *v*/*v*; solvent B), and acetonitrile (solvent C). The gradient was as follows: 3% B and 97% C, 0–5 min; 9% B and 91% C, 5–15 min; 16% B and 84% C, 15–30 min; 33% B and 67% C, 30–33 min; 90% A and 10% B, 33–35 min; 90% B and 10% C, 32–42 min; 3% B and 97% C. Flow rate was fixed at 0.2 mL/min, and signals were monitored at 245, 280, 320, and 550 nm. MS analyses were conducted in negative ion mode, scanning 50–2000 *m*/*z*. Nitrogen served as the nebulizing gas and helium as the damping gas. Ion source parameters included 5.0 kV spray voltage, 90 V capillary voltage, and 350 °C temperature. Data were processed using software MS Workstation (Varian Medical Systems, Inc., Palo Alto, CA, USA v6.9 licensed by VARIAN) [14].

### 2.10. Antioxidant Determination by DPPH Method and ABTS Radical Scavenging Assay

Antioxidant activity of crude, extracted, fractionated, and semi-purified samples (PDB120 and PDB+LP144) were assessed using DPPH and ABTS assays.

For DPPH, a 1 mM Trolox standard was used to prepare the calibration curve. Samples (100 µL) were mixed with 100 µL DPPH solution (0.1 mM) and incubated at 30 °C in the dark for 30 min. Absorbance was measured at 517 nm. Results are expressed as µmol Trolox equivalents (µmol TE; 20–400 µM calibration curve) and as percentage inhibition (Equation (2)).

For ABTS, the radical cation was generated by mixing 7 mM ABTS with 2.45 mM potassium persulfate and incubating in the dark for 16 h. Before analysis, the solution was diluted with ethanol to an absorbance of 0.700 ± 0.02 at 734 nm. Samples (10 µL) were added to 1 mL of ABTS working solution, incubated for 6 min at room temperature, and measured at 734 nm. Antioxidant activity was expressed as µmol TE (based on the same calibration curve) and as percentage inhibition (Equation (2)). Measurements were performed in triplicate, and results were analyzed in Microsoft Excel.

(2) $$Inhibition\left(\%\right)=\frac{Abs\;control-Abs\;sample}{Abs\;control}\times 100$$

### 2.11. Statistical Analysis

All experiments were performed in triplicate (*n* = 3), and results were expressed as mean ± standard deviation (SD). Data were analyzed by one-way ANOVA, followed by Tukey’s post hoc test to determine significant differences (*p* < 0.05). Statistical analyses were performed using Origin Pro 2023 software.

## 3. Results and Discussion

### 3.1. Evaluation of Antimicrobial Activity of Extracellular Compounds from the Crude Samples

The antimicrobial activity of extracellular compounds obtained from crude samples produced under submerged fermentation (96 h) was assessed using the disk diffusion method against Gram-negative and Gram-positive bacteria, as well as against yeast. The inhibition zones observed are summarized in Table 1.

To determine whether any antimicrobial effects originated from the culture media themselves, uninoculated PDB and PDB+LP (lemon peel) samples were evaluated against *E. coli* and *S. aureus*. Minimal inhibitory activity was observed for the Gram-positive bacterium in both conditions. However, for *E. coli*, a small inhibition zone (0.45 ± 0.3 mm) was detected. Although this effect was minor (<1 mm), it is consistent with the intrinsic chemical profile of citrus peel, which contains phenolic acids, flavonoids, and essential oils with reported antimicrobial effects [16]. Extracts derived from citrus by-products are generally more effective against Gram-negative than Gram-positive bacteria [16].

Clear antibacterial activity against *E. coli* and *S. aureus* was observed in inoculated fermentation samples of *P. crustosum* cultivated in both media. For *E. coli*, the largest inhibition zone (5.17 ± 0.25 mm) occurred in PDB+LP, indicating that lemon peel significantly enhanced the synthesis or secretion of metabolites active against this Gram-negative pathogen. Notably, this inhibition zone exceeded that produced by the positive control (streptomycin, 2 mg/mL), underscoring the high potency of the crude extract and suggesting its potential as a microbial control alternative.

In contrast, against *S. aureus*, the highest inhibition zone (4.35 ± 0.15 mm) was obtained in PDB without co-substrate. This indicates that lemon peel supplementation does not necessarily enhance the biosynthesis of compounds active against Gram-positive bacteria and may, in fact, shift metabolic pathways toward metabolites with greater specificity for Gram-negative organisms. These findings reinforce the notion that substrate composition modulates fungal metabolic responses, shaping the spectrum of antimicrobial compounds produced [17].

No antimicrobial activity against *C. albicans* was detected under either fermentation condition. This absence contrasts with the antibacterial activity observed and suggests that the metabolites produced under these conditions are more selective for bacteria than for yeasts. The intrinsic resistance of *C. albicans*—due to its thick cell wall composed of β-glucans and chitin—likely contributes to this outcome [18]. Additionally, many effective antifungal agents, such as fluconazole, target ergosterol biosynthesis, a mechanism not reflected in the metabolites produced by *P. crustosum* OR889307 under the evaluated conditions [19].

Although some *Penicillium* strains, such as *P. meleagrinum*, have demonstrated antifungal activity against *C. albicans*, these effects were reported under markedly different fermentation conditions—specifically, longer fermentation times (13 days), biomass-derived extracts, and stress-induced metabolite profiles [20]. Such differences highlight the importance of fermentation duration and medium composition, as minimal media have been associated with enhanced production of antifungal metabolites. Thus, the absence of antifungal activity in the present study is plausibly linked to the specific fermentation parameters employed.

### 3.2. Effects of Fermentation Time and Co-Substrate on the Production of Antimicrobial Compounds

**Figure 1:**
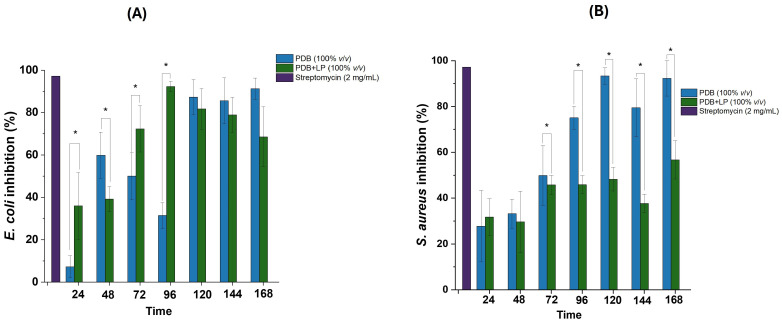
Antimicrobial activity of *Penicillium crustosum OR889307* supernatant produced under submerged fermentation at different periods of time (h) in Potato Dextrose Broth (PDB) and Potato Dextrose Broth with 2% (dry weight) lemon peel (PDB+LP) against *Escherichia coli* (**A**) and *Staphylococcus aureus* (**B**). The positive control was streptomycin (2 mg/mL). Data are shown as means ± standard error (*n* = 3). Significant statistical differences in a pair of means (*p* < 0.05) are represented by an asterisk (*).

The influence of fermentation time on the production of antimicrobial compounds by *P. crustosum* OR889307 was evaluated across a period of 0–168 h (Figure 1A,B), focusing on *E*. *coli* and *S*. *aureus*, given the absence of antifungal effects against *C*. *albicans*.

For *E. coli* (Figure 1A), fermentation time had a significant effect (α = 0.05) on the production of extracellular antimicrobial metabolites in both media. The highest growth inhibition (89.03%) occurred at 96 h in PDB+LP, with no significant increase thereafter (120–168 h). This progressive enhancement suggests that metabolite biosynthesis follows a typical secondary metabolism pattern, where production intensifies during mid-to-late fermentation stages [21]. The co-substrate also significantly influenced antimicrobial output. Lemon peel likely promoted sustained metabolite synthesis by providing readily assimilable nutrients and precursors such as phenolics, which may act synergistically against Gram-negative bacteria. *Penicillium* species are also known to synthesize terpenoids such as limonene and essential oils with antibacterial properties [22], which may be enhanced in the presence of lemon peel-derived compounds.

In the case of *S. aureus* (Figure 1B), fermentation time and co-substrate presence also had significant effects; however, the behavior differed from that observed for *E. coli*. The maximum inhibition (93.31 ± 3.57%) occurred in PDB at 120 h—substantially higher than the maximum inhibition observed with PDB+LP (56.79 ± 8.38% at 168 h). Interestingly, this peak in antibacterial activity coincided with the highest antioxidant activity at the same time point (120 h), suggesting that specific secondary metabolites with dual antioxidant–antimicrobial properties were predominant under these conditions. Variation in metabolite profiles between PDB and PDB+LP further reinforces the role of medium composition in modulating fungal metabolic pathways.

Based on these results, two samples were selected for further characterization: PDB+LP at 144 h (PDB+LP144) for activity against *E. coli* and PDB at 120 h (PDB120) for activity against *S. aureus*.

### 3.3. Antimicrobial Activity of Crude, Extracted, Fractionated, and Semi-Purified Samples Against E. coli, S. aureus, MRSA, and P. aeruginosa

The selected samples (PDB120 and PDB+LP144), which exhibited antimicrobial activity against *S. aureus* and *E. coli*, were further examined for their efficacy against methicillin-resistant *Staphylococcus aureus* (MRSA) and *Pseudomonas aeruginosa*. The assessment included the aqueous and organic phases obtained after ethyl acetate extraction, as well as the >10 kDa and <10 kDa molecular weight fractions. In addition, their corresponding semi-purified samples (semi-purified PDB120 and semi-purified PDB+LP144) were evaluated.

**Figure 2:**
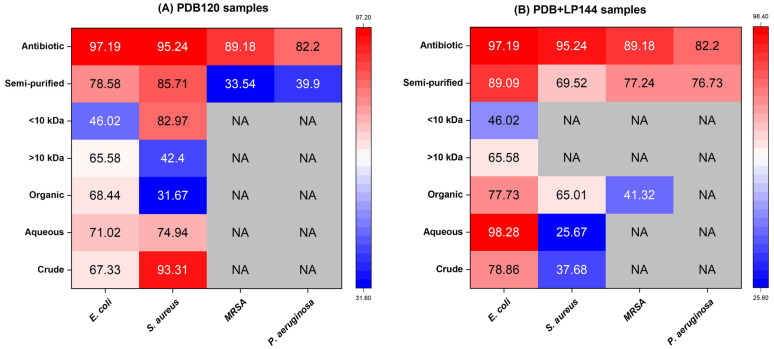
Heatmap representation of growth inhibition (%) of *Escherichia coli*, *Staphylococcus aureus*, methicillin-resistant *S. aureus* (MRSA), and *Pseudomonas aeruginosa* by different extracts obtained from submerged fermentation in Potato Dextrose Broth (PDB120) (**A**) and Potato Dextrose Broth with 2% (dry weight) lemon peel (PDB+LP144) (**B**). Streptomycin (2 mg/mL) was used as a positive control. Color intensity represents the percentage of growth inhibition. NA indicates no antimicrobial activity under the tested conditions. This graph was generated using Origin Pro 2023 (OriginLab Corporation, USA) and row normalization (maximum = 1).

The results, summarized in Figure 2A,B, demonstrate distinct inhibitory patterns across all treatments. The heat map clearly highlights variations in antimicrobial potency, with the co-substrate–supplemented sample (PDB+LP144) displaying improved activity against both Gram-positive and Gram-negative bacteria compared with the PDB120 sample. These findings indicate that lemon peel supplementation promotes the formation or concentration of bioactive metabolites with broader antimicrobial efficacy.

The crude extract from PDB+LP144 exhibited stronger inhibition against *E. coli* (78.86 ± 8.34%) than PDB120 (67.33 ± 13.2%), indicating that the addition of lemon peel as a co-substrate enhances the production of metabolites—particularly those active against Gram-negative bacteria. In contrast, the PDB120 crude extract showed significantly higher activity against *S. aureus* (93.31 ± 1.57%) compared with PDB+LP144 (37.68 ± 4.04%), suggesting that lemon peel supplementation is less effective for promoting the synthesis of compounds targeting Gram-positive bacteria [16]. Neither crude extract exhibited inhibitory activity against MRSA or *P. aeruginosa*.

Following ethyl acetate extraction, the aqueous phase of PDB+LP144 displayed the highest inhibitory activity against *E. coli* (98.28 ± 0.91%), exceeding that of streptomycin. The organic phase of PDB+LP144 also demonstrated substantial inhibition (77.73 ± 5.94%), surpassing the activity of the corresponding organic phase from PDB120 (68.44 ± 4.30%), thus indicating the production of lipophilic antimicrobial metabolites [8]. For *S. aureus*, the aqueous phase of PDB120 showed higher activity (74.94 ± 7.07%) than its organic phase (31.67 ± 5.55%), once again underscoring the relevance of polar compounds in the inhibition of Gram-positive bacteria. None of the extracted phases showed measurable activity against MRSA or *P. aeruginosa*.

For *E. coli*, both >10 kDa fractions retained moderate inhibition (65.58 ± 17.31% for PDB120 and 65.58 ± 7.31% for PDB+LP144), whereas their corresponding <10 kDa fractions exhibited reduced activity (46.02 ± 0.56%). Conversely, in *S. aureus*, the <10 kDa fraction of PDB120 displayed notably higher inhibition (82.97 ± 0.19%) than the >10 kDa fraction (42.40 ± 5.94%), suggesting that low-molecular-weight metabolites play a central role in inhibiting this Gram-positive pathogen. Fractions from PDB+LP144 showed minimal activity against *S. aureus*. No inhibitory effects were detected against MRSA or *P. aeruginosa* in any of the fractionated samples.

Semi-purified samples exhibited a marked increase in antimicrobial activity under both culture conditions. For instance, semi-purified PDB+LP144 demonstrated higher inhibition against *E. coli* (89.09 ± 2.39%) compared with its crude or fractionated forms. In the case of *S. aureus*, semi-purified PDB120 maintained the activity pattern observed in the crude and extracted samples, achieving 85.71 ± 2.45% inhibition. Notably, the semi-purified extracts were the only samples exhibiting activity against MRSA and *P. aeruginosa*. PDB+LP144 displayed higher inhibition against both pathogens (77.24 ± 8.62% for MRSA and 76.73 ± 7.12% for *P. aeruginosa*) than PDB120 (33.54 ± 4.58% and 39.90 ± 5.74%, respectively). These results suggest that co-substrate fermentation promotes the synthesis of specialized metabolites capable of overcoming resistance mechanisms in these clinically challenging pathogens.

### 3.4. Determination of Minimum Inhibitory Concentration (MIC) of Semi-Purified Samples

The minimum inhibitory concentration (MIC) was determined to further evaluate the antimicrobial efficacy of the semi-purified samples: PDB120 against *S. aureus* and PDB+LP144 against *E. coli*, MRSA, and *P. aeruginosa*. Streptomycin (2 mg/mL) was used as the standard antibiotic control.

The semi-purified samples were rehydrated in sterile distilled water to obtain concentrations ranging from 500 to 0.6 µg/mL. The MIC value obtained for *S. aureus* using the PDB120 semi-purified extract was 250 µg/mL, which is higher than the MIC of 50 µg/mL previously reported for an *Aspergillus flavus* MTCC 13062 extract against *S. aureus* [15]. For *E. coli*, the PDB+LP144 semi-purified sample exhibited an MIC of 125 µg/mL, comparable to reports describing MIC values of approximately 50 µg/mL for *A. flavus* extracts against this Gram-negative pathogen [15].

**Figure 3:**
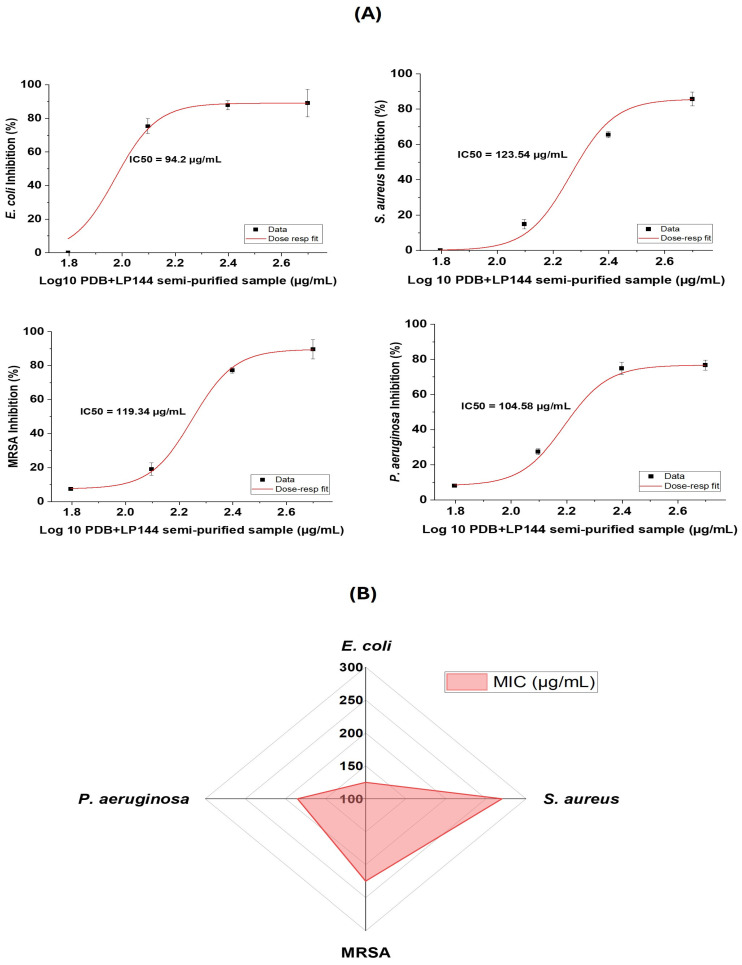
(**A**) Dose–response curves of bacterial growth inhibition by the PDB+LP144 semi-purified sample against *E. coli*, *S. aureus*, MRSA: Methicillin-resistant *Staphylococcus aureus*, and *P. aeruginosa*. The *IC_50_* values were calculated from the fitted curves. (**B**) Radial plot of MIC values of semi-purified PDB+LP144 extract against bacterial strains. Streptomycin (2 mg/mL) was used as a standard antibiotic control, with the MIC values of 1.0, 1.2, 1.6, and >2.0 µg/mL against *E. coli*, *S. aureus*, *P. aeruginosa,* and SARM, respectively.

In addition, the MIC values determined for MRSA and *P. aeruginosa* from the PDB+LP144 semi-purified sample were 225 µg/mL and 185 µg/mL, respectively. These MIC values are summarized in the radial plot (Figure 3B), which highlights the greater susceptibility of *E. coli* and *S. aureus* relative to MRSA and *P. aeruginosa*. This trend is further supported by the dose–response curves, which yielded *IC_50_* values of 94.2 µg/mL for *E. coli*, 123.5 µg/mL for *S. aureus*, 119.3 µg/mL for MRSA, and 104.9 µg/mL for *P. aeruginosa* (Figure 3A). These results align with previous studies reporting MIC values of approximately 200 µg/mL for mycelium-derived *A. flavus* extracts against these pathogens [23].

It is important to note that the semi-purification strategy used in this study did not involve additional solvent-based steps or extraction from the mycelial biomass. The active compounds were produced extracellularly, thereby reducing the need for solvent-intensive biomass extraction. This highlights the potential of extracellular bioactive metabolites as a sustainable and scalable source of antimicrobial agents.

### 3.5. Identification of Antimicrobial Compounds Using RP-HPLC-ESI-MS

Crude extracts from PDB120 and PDB+LP144, recovery extracts (organic and aqueous phases), fractionated samples (<10 kDa and >10 kDa), semi-purified samples, and biomass were analyzed by RP-HPLC-ESI-MS (Table 2). Tentative compound identification was assigned based on retention time, accurate mass (*m*/*z*), and comparison with reported MS/MS fragmentation patterns. The corresponding chromatograms are provided in the Supplementary Material (Figures S1 and S2). The metabolite profiling of *P. crustosum* revealed a clear influence of cultivation conditions on the presence and distribution of specific compounds (Tables S1 and S2). Certain metabolites, such as ferulic acid 4-O-glucoside and quercetin derivatives, were consistently detected across several treatments, indicating that they represent major secondary metabolites produced under multiple conditions. Both compounds are well-documented for their health-promoting properties, particularly antioxidant and anti-inflammatory activities [24].

In contrast, several metabolites were detected only under specific extraction or processing conditions. For example, bisdemethoxycurcumin appeared exclusively in the organic phase of PDB120, while spinacetin glycosides were identified only in the semi-purified fraction of PDB+LP144. These differences can be attributed to variations in polarity, molecular weight distribution, and compound solubility, which govern the partitioning of metabolites into aqueous, organic, or size-exclusion fractions. Moreover, the presence of a co-substrate in PDB+LP144 likely modulated fungal metabolic pathways, promoting the biosynthesis of flavonoids and lignans that were absent in the PDB120 condition [7].

Bioactive compounds such as 3,4-diferuloylquinic acid and caffeic acid derivatives indicated the presence of both hydroxycinnamic and methoxycinnamic acids in the fermentation extracts. These compounds are known as antimicrobial agents, particularly against *S. aureus* [24]. This result is consistent with the activity observed in the PDB120 crude extract, which showed strong inhibition of *S. aureus* (93.31 ± 1.57%), moderate activity against *E. coli*, and no detectable activity against MRSA or *P. aeruginosa*. The methoxycinnamic acids identified in the PDB120 crude sample have been reported to disrupt cell-wall biosynthesis in *S. aureus* [24].

In contrast, the PDB+LP144 extracts—which contained compounds such as ferulic acid 4-O-glucoside and quercetin 3-O-xylosyl-glucuronide—exhibited markedly greater antimicrobial activity against *E. coli* than PDB120, underscoring their high bioactive potential against Gram-negative bacteria [7].

Assessment of the aqueous phase obtained after ethyl acetate extraction revealed increased inhibition of *E. coli* and *S. aureus* in PDB120, whereas activity against *P. aeruginosa* and MRSA decreased. Notably, this phase contained 3,4-DHPEA-EA (3,4-dihydroxyphenylethanol-ethyl ester), a compound previously identified in *Aspergillus niger* extracts and known for its antimicrobial activity against *S. aureus* [14]. In the organic phase of PDB120, inhibition of *E. coli* was retained, although reduced activity was observed for *S. aureus*. This phase contained bisdemethoxycurcumin, a curcumin derivative associated with membrane disruption in Gram-negative bacteria [25].

Interestingly, the aqueous phase of the PDB+LP144 extract exhibited the highest inhibition of *E. coli*, while showing weak activity against *S. aureus*, suggesting that the water-soluble components produced under co-substrate conditions preferentially target Gram-negative bacteria. Conversely, the organic phase of PDB+LP144 demonstrated strong activity against *S. aureus* and moderate inhibition of MRSA, indicating that this fraction contains bioactive metabolites capable of targeting both Gram-positive and antibiotic-resistant pathogens [24].

Further characterization of the PDB120 fractions revealed that the >10 kDa fraction displayed reduced activity against both *E. coli* and *S. aureus*, suggesting that larger molecules contribute less to the antimicrobial profile. In contrast, the <10 kDa fraction showed enhanced activity against *S. aureus*, indicating that low-molecular-weight metabolites play a more significant role in the observed inhibition. A similar pattern was evident in the >10 kDa and <10 kDa fractions from PDB+LP144, reinforcing the contribution of small molecules to antimicrobial activity [26].

The semi-purified PDB120 extract, obtained using Amberlite XAD16 column chromatography, displayed the strongest antimicrobial activity among all PDB120-derived fractions, particularly against *S. aureus*, *E. coli*, *P. aeruginosa*, and MRSA. This suggests that the semi-purification process effectively enriched metabolites with broader antimicrobial potential. Quercetin 3-O-xylosyl-glucuronide, a flavonol with well-documented antioxidant and antimicrobial activities, was identified in this fraction [7]. Secoisolariciresinol, a lignan, was also detected; lignans are widely recognized for their antimicrobial effects, especially against Gram-positive bacteria, supporting the strong inhibitory activity observed against *S. aureus* and MRSA, as well as the moderate activity against *P. aeruginosa* [27,28].

The semi-purified PDB+LP144 sample exhibited superior antimicrobial activity across all tested pathogens. Its high efficacy suggests that fermentation in the presence of lemon peel as a co-substrate substantially enhanced the production of bioactive metabolites capable of broad-spectrum antimicrobial activity. Key compounds identified in this fraction included flavonols and flavanones such as quercetin 3-O-xylosyl-glucuronide and naringin. Additionally, methoxycinnamic derivatives such as spinacetin 3-O-glucosyl-(1→6)-[apiosyl(1→2)]-glucoside and ferulic acid 4-O-glucoside were detected. These compounds are known for their antimicrobial and antioxidant properties and likely contributed to the enhanced activity observed in the semi-purified PDB+LP144 sample [27,28].

Interestingly, ferulic acid 4-O-glucoside, naringin, and quercetin 3-O-xylosyl-glucuronide were detected in both control and lemon peel-supplemented fermentations. Because these compounds were produced even in the absence of lemon peel, their presence is attributed to the intrinsic metabolic activity of *P. crustosum*, rather than to direct extraction from the plant co-substrate.

Conversely, bisdemethoxycurcumin and spinacetin glycosides were detected exclusively in the lemon peel-supplemented cultures, suggesting that these metabolites likely originated through biotransformation of lemon peel-derived precursors [11].

### 3.6. Influence of Lemon Peel Co-Substrate on Antioxidant Activity (DPPH and ABTS) and Metabolite Composition

The antioxidant activity of the crude extract, aqueous phase, organic phase, >10 kDa fraction, <10 kDa fraction, and the semi-purified samples obtained from *P. crustosum* OR889307 cultivated in standard PDB medium (PDB120) or PDB supplemented with 2% (*w*/*v*) lemon peel (PDB+LP144) was evaluated using DPPH radical scavenging and ABTS assays, expressed as µmol TE (Trolox equivalents) (Table 3). A control sample consisting of PDB medium supplemented with lemon peel but without fungal inoculation was included in both assays to account for the intrinsic antioxidant activity contributed solely by the citrus substrate. All values reported in Table 3 represent corrected antioxidant activity after subtracting the contribution of the control (31.6 ± 2.45 µmol TE for DPPH and 29.1 ± 0.98 µmol TE for ABTS), ensuring that the results reflect only the metabolites produced during fermentation.

The highest DPPH values were recorded in the crude extract, aqueous fraction, and >10 kDa fraction of PDB120 (80–86 µmol TE), indicating that high-molecular-weight antioxidant compounds were predominant in this condition. The crude extract also exhibited the strongest inhibition (70.52 ± 1.58%), followed by the organic phase (79.49 ± 2.05%). These results align with previous reports of *A. unguis* cultivated in potato dextrose broth, which achieved 65% radical scavenging activity under shake conditions [29], and *Penicillium granulatum*, which displayed 72 ± 0.2% DPPH inhibition using dextrose as a carbon source under stationary fermentation for 10 days [30]. RP-HPLC-ESI-MS analysis supports this observation, as these fractions predominantly contain 3,4-dihydroxyphenylethanol-elenolic acid and bisdemethoxycurcumin, both recognized for strong radical-scavenging activity.

In contrast, DPPH inhibition in PDB+LP144 samples was generally lower (25–55 µmol TE), with the highest activity observed in the <10 kDa fraction (55.65 ± 3.68%). This suggests a metabolic shift toward the production of smaller antioxidant molecules in the presence of the co-substrate. This trend is consistent with RP-HPLC-ESI-MS data showing the presence of ferulic acid 4-O-glucoside, *p*-coumaric acid ethyl ester, and secoisolariciresinol—phenolics known for high reactivity toward free radicals. The presence of pectin-derived oligosaccharides characteristic of lemon peel likely influences fungal carbon utilization and reshapes the antioxidant metabolite profile [31].

The ABTS assay revealed a contrasting distribution, with the semi-purified fraction of PDB+LP144 exhibiting the strongest activity (283.95 ± 2.17 µmol TE), followed by the organic fraction (138.4 ± 33.61 µmol TE). In comparison, PDB120 samples showed moderate ABTS activity, with the semi-purified fraction achieving the highest response within this group (50.11 ± 3.67%). These values are comparable to those reported for mycelial extracts of *P. expansum* (72.54 ± 0.05%) [32].

These results suggest that although PDB120 contains antioxidant compounds detectable by ABTS, their concentration is considerably lower than that produced under lemon peel co-fermentation. The enhanced antioxidant activity observed under co-substrate conditions may result from the presence of polyphenolics naturally present in lemon peel (such as naringin, ferulic acid derivatives, and various flavonoids), which act as radical scavengers and may additionally stimulate fungal biosynthesis of antioxidant metabolites [16].

Differences between the two media can be attributed to the presence and diversity of specific phenolic compounds generated during co-fermentation. RP-HPLC-ESI-MS profiling confirmed that fractions exhibiting high ABTS activity in PDB+LP144 contained naringin, quercetin 3-O-xylosyl-glucuronide, and spinacetin glycosides, all of which have strong ABTS reactivity due to their substitution patterns and enhanced electron-donating capacity [33].

These findings demonstrate that ABTS is more sensitive to the metabolites enriched during lemon-peel-assisted fermentation, whereas PDB120 maintains a baseline antioxidant capacity associated with polymeric or high-molecular-weight metabolites, consistent with the detection of bisdemethoxycurcumin among its constituents [25].

*Penicillium* species are well-known producers of phenolic and flavonoid compounds through secondary metabolism, contributing significantly to their antioxidant properties. For example, *P. coprophilum* produces roquefortine [2], and *P. setosum* generates vanillic acid along with flavonols such as kaempferol, quercetin, and luteolin [34]. These fungi also synthesize other antioxidant metabolites, including vitamin C and carotenoid pigments, further supporting their relevance as sources of antioxidant bioactive compounds [35].

## 4. Conclusions

This study demonstrates, for the first time, that the incorporation of lemon peel as a co-substrate significantly enhances the extracellular production of antimicrobial and antioxidant metabolites by *P. crustosum* OR889307 under submerged fermentation. This finding is consistent with the observed increase in bioactivity across all tested bacterial strains, particularly *E. coli*, MRSA, and *P. aeruginosa*, which exhibited the highest susceptibility to semi-purified extracts. These antimicrobial effects were further supported by the MIC and *IC_50_* profiles, which collectively indicate that the lemon peel-supplemented fermentation system promotes the formation of bioactive molecules with broad-spectrum inhibitory potential.

Chemical profiling via RP-HPLC-ESI-MS revealed the presence of phenolic and flavonoid derivatives such as quercetin 3-O-xylosyl-glucuronide and ferulic acid 4-O-glucoside. The detection of these compounds aligns with the observed antioxidant and antimicrobial activities, reinforcing the role of lemon peel as a functional inducer of secondary metabolite synthesis in filamentous fungi.

Beyond its biological relevance, this work highlights a sustainable and scalable bioprocessing strategy. The use of an agro-industrial residue as a metabolic enhancer not only reduces substrate costs but also contributes to circular bioeconomy principles by valorizing fruit-processing byproducts. Moreover, the extracellular nature of the bioactive metabolites eliminates the need for solvent-intensive biomass extraction, simplifying downstream processing and improving environmental compatibility.

Overall, the integration of co-substrate supplementation with submerged fermentation and minimal semi-purification steps establishes an efficient platform for producing natural antimicrobial and antioxidant agents. These findings underline the potential application of *P. crustosum*-derived metabolites in food preservation and safety, as well as in the development of functional ingredients with health-promoting properties. Future work should focus on compound isolation, structural elucidation, and evaluation in food matrices to fully validate their technological and biological applicability.

## Figures and Tables

**Table 1:** Antimicrobial activity of crude samples of *Penicillium crustosum* OR889307 evaluated by a zone of inhibition (measured in mm, ±SD) against pathogenic microorganisms.

Experiment	Pathogenic Strains		
	*E. coli*	*S. aureus*	*C. albicans*
PDB (without fungal inoculation)	n.d.	n.d.	n.d.
PDB+LP (without fungal inoculation)	0.45 ± 0.3	0.07 ± 0.00	n.d.
PDB	1.73 ± 0.03	4.35 ± 0.15	n.d.
PDB+LP	5.17 ± 0.25	n.d.	n.d.
Streptomycin	4.44 ± 0.56	4.43 ± 0.56	-
Fluconazole	-	-	3.97 ± 1.08
Negative control	n.d.	n.d.	n.d.

Data are presented as means ± standard error (*n* = 3); Streptomycin (2 mg/mL), Fluconazole (2 mg/mL); Negative control = sterile water. The n.d. means “not detected”.

**Table 2:** Tentative identification of bioactive compounds in submerged fermentation samples by RP-HPLC-ESI-MS. Compounds were identified based on retention time, molecular ion (*m*/*z*), and characteristic MS/MS fragmentation patterns.

Sample	Fraction	ID	Retention Time (min)	Mass (*m*/*z*)/[M-H]^−^	Compound	Chemical Class
PDB120	Crude	1	5.014	826.5	-	-
	Aqueous phase	2	5.015	826.7	-	-
		3	5.208	376.9	3,4-dihydroxyphenylethanol-elenolic acid (3,4-DHPEA-EA)	Tyrosols
		4	7.82	602	-	-
	Organic phase	5	5.144	376.8	3,4-dihydroxyphenylethanol-elenolic acid	Tyrosols
		6	11.202	306.7	Bisdemethoxycurcumin	Curcuminoid
		7	32.369	722.9	1-O-Sinapoyl-2-O-feruloyl gentibiose	Methoxycinnamic acid
	>10 kDa	8	4.659	376.9	3,4-dihydroxyphenylethanol-elenolic acid	Tyrosol
	<10 kDa	9	4.456	356.7	Ferulic acid 4-O-glucoside	Methoxycinnamic acid
	Semi-purified	10	5.283	664.7	Naringin 6′-malonate	Flavonone
		11	18.89	364.8	Secoisolariciresinol	Lignan
		12	23.543	356.6	Ferulic acid 4-O-glucoside	Methoxycinnamic acid
		13	32.992	364.8	Secoisolariciresinol	Lignan
		14	36.245	608.8	Quercetin 3-O-xylosyl-glucuronide	Flavonol
PDB+LP144.	Control *	15	4.086	356.7	Ferulic acid 4-O-glucoside	Methoxycinnamic acid
		16	40.457	292.6	Caffeoyl aspartic acid	Hydroxycinnamic acids
		17	5.501	190.9	p-Coumaric acid ethyl ester	Hydroxycinnamic acids
		18	22.426	364.8	Secoisolariciresinol	Lignan
		19	36.245	608.8	Quercetin 3-O-xylosyl-glucuronide	Flavonol
	Crude	20	5.154	544.8	3,4-Diferuloylquinic acid	Methoxycinnamic acid
		21	20.171	365.0	Secoisolariciresinol	Lignan
		22	27.065	356.8	Ferulic acid 4-O-glucoside	Methoxycinnamic acid
		23	35.041	723.0	1-O-Sinapoyl-2-O-feruloyl gentiobiose	Methoxycinnamic acid
	Aqueous phase	24	5.007	190.9	p-Coumaric acid ethyl ester	Hydroxycinnamic acids
		215	26.082	356.9	Ferulic acid 4-O-glucoside	Methoxycinnamic acid
		26	34.975	608.9	Quercetin 3-O-xylosyl-glucuronide	Flavonol
	Organic phase	27	4.660	190.9	p-Coumaric acid ethyl ester	Hydroxycinnamic acids
		28	31.517	608.7	Quercetin 3-O-xylosyl-glucuronide	Flavonol
		29	35.458	292.6	Caffeoyl aspartic acid	Hydroxycinnamic acids
		30	21.919	356.8	Ferulic acid 4-O-glucoside	Methoxycinnamic acid
	>10 kDa	31	5.071	368.9	Sesaminol	Lignan
		32	7.476	364.9	Secoisolariciresinol	Lignan
		33	37.440	608.8	Quercetin 3-O-xylosyl-glucuronide	Flavonol
	<10 kDa	34	5.221	190.9	p-Coumaric acid ethyl ester	Hydroxycinnamic acids
		35	11.688	356.7	Ferulic acid 4-O-glucoside	Methoxycinnamic acid
		36	21.083	365.0	Secoisolariciresinol	Lignan
		37	26.281	357.0	Ferulic acid 4-O-glucoside	Methoxycinnamic acid
		38	35.974	608.7	Quercetin 3-O-xylosyl-glucuronide	Flavonol
	Semi-purified	39	5.324	340.7	Caffeic acid 4-O-glucoside	Hydroxycinnamic acids
		40	8.578	356.5	Ferulic acid 4-O-glucoside	Lignan
		41	31.586	808.0	Spinacetin 3-O—glucosyl-(1-6)-[apiosyl(1-2)]-glucoside	Methoxyflavonol
		42	35.743	578.7	Naringin	Flavonone
		43	37.851	608.7	Quercetin 3-O-xylosyl-glucuronide	Flavonol
		44	43.283	794.0	-	-

Fractions <10 kDa and >10 kDa refer to molecular weight cut-off filtrates obtained by ultrafiltration. Control * means PDB+LP 2% (*w*/*v*) without inoculation.

**Table 3:** Antioxidant activity of PDB120 and PDB+LP144 samples determined by DPPH and ABTS radical scavenging assays, expressed as % inhibition and µmol Trolox.

	DPPH Radical Scavenging Assay				ABTS Radical Scavenging Assay			
Fractions	PDB120 Samples		PDB+LP144 Samples		PDB120		PDB+LP144 Samples	
	% Inhibition	µmol TE	% Inhibition	µmol TE	% Inhibition	µmol TE	% Inhibition	µmol TE
Crude	70.3 ± 1.58	84.91 ± 2.58	49.87 ± 2.58	46.31 ± 1.02	21.48 ± 0.97	50.79 ± 11.01	18.49 ± 0.56	24.58 ± 7.99
Organic phase	70.59 ± 2.05	85.42 ± 5.56	34.92 ± 1.51	25.9 ± 0.36	20.18 ± 4.05	32.95 ± 7.65	38.51 ± 5.02	138.54 ± 33.21
Aqueous phase	67.43 ± 1.47	80.01 ± 1.36	37.65 ± 0.87	29.72 ± 2.98	8.41 ± 1.02	7.75 ± 0.05	11.33 ± 1.65	14.79 ± 0.93
>10 kDa	70.52 ± 5.08	85.34 ± 6.98	52.76 ± 4.05	50.63 ± 1.45	17.72 ± 1.06	37.5 ± 3.81	20.18 ± 2.54	49.45 ± 12.37
<10 kDa	37.51 ± 0.94	29.58 ± 1.25	55.65 ± 3.68	54.92 ± 0.99	26.48 ± 1.58	32.95 ± 7.65	12.77 ± 0.94	21.79 ± 1.33
Semi-purified	33.12 ± 1.03	22.31 ± 2.48	43.54 ± 2.78	54.72 ± 0.97	50.11 ± 3.67	194.91 ± 32.8	68.43 ± 5.31	238.95 ± 2.17
Control *	-	-	45.98 ± 1.05	31.6 ± 2.45	-	-	54.93 ± 3.21	29.1 ± 0.98

Values are expressed as mean ± standard deviation (*n* = 3). Control * corresponds to the experiment using extracts of PDB120 and PDB+LP144 without fungal inoculation.

## Data Availability

The original contributions presented in this study are included in the article/Supplementary Materials. Further inquiries can be directed to the corresponding author.

## Supplementary Materials

The following supporting information can be downloaded at https://www.mdpi.com/article/10.3390/foods15020348/s1: Figure S1. RP-HPLC profiles of PDB120 Crude and Semi-purified extracts. Figure S2. RP-HPLC profiles of PDB+LP144 Crude and Semi-purified extracts. Table S1. Retention times and tentative identification of compounds detected by RP-HPLC in PDB120 crude and semi-purified extracts. Table S2. Retention times and tentative identification of compounds detected by RP-HPLC in PDB+LP144 crude and semi-purified extracts.
