# Supplementary material for: Production of Antimicrobial and Antioxidant Metabolites by Penicillium crustosum Using Lemon Peel as a Co-Substrate in Submerged Fermentation
Source: Foods. 2026 Jan 18;15(2):348. doi: 10.3390/foods15020348 (PMC12840410; doi:10.3390/foods15020348)
Supplement: Supplementary file 1 [file foods-15-00348-s001.zip › foods-3989038-supplementary.pdf]

Supplementary material

Production of Antimicrobial and Antioxidant Metabolites by *Penicillium crustosum* Using Lemon Peel as a Co-Substrate in Submerged Fermentation

Arely Núñez-Serrano[1], Refugio B. García-Reyes[1], Juan A. Ascasio-Valdés[2], Cristóbal N. Aguilar-González[2], Alcione García-González[1*]

[1] Facultad de Ciencias Químicas, Universidad Autónoma de Nuevo León (UANL), Av. Universidad S/N, Cd. Universitaria, San Nicolás de los Garza 66455, Nuevo León, Mexico; arely.nunezsrr@uanl.edu.mx (A.N.-S.); refugio.garciary@uanl.edu.mx (R.B.G.-R.)

[2]Bioprocesses and Bioproducts Research Group, Food Research Department, School of Chemistry, Universidad Autónoma de Coahuila, Unidad Saltillo, Saltillo 25280, Coahuila, Mexico; alberto_ascaciovaldes@uadec.edu.mx (J.A.A.-V.); cristobal.aguilar@uadec.edu.mx (C.N.A.-G.)

[*]Correspondence: alcione.garciagn@uanl.edu.mx

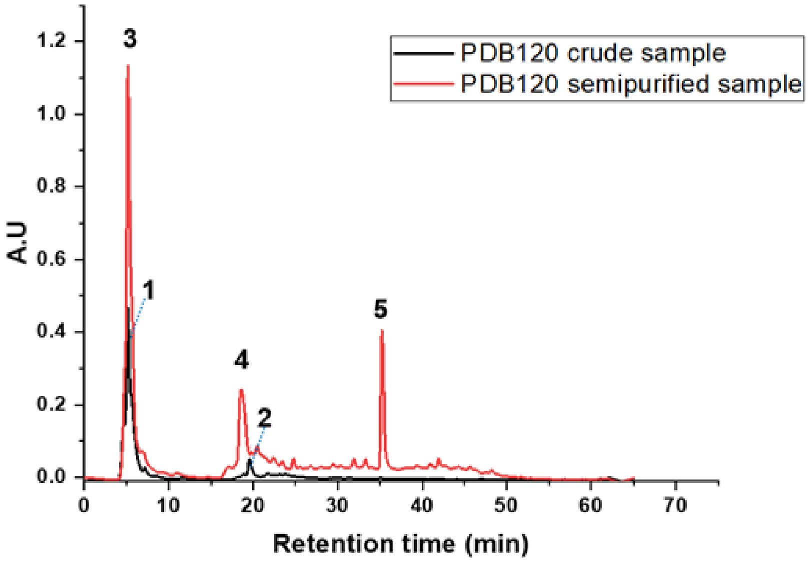

Figure S1. RP-HPLC profiles of PDB120 Crude and Semi-purified extracts

Table S1. Retention times and tentative identification of compounds detected by RP-HPLC in PDB120 crude and semi-purified extracts.

Sample		Retention time (min)	Compound
Crude	1	5.007	Ferulic acid 4-O-glucoside
	2	20.17	Secoisolariciresinol
Semi-purified	3	5.014	Ferulic acid 4-O-glucoside
	4	18.89	Secoisolariciresinol
	5	36.2	Quercetin 3-O-xylosyl-glucuronide

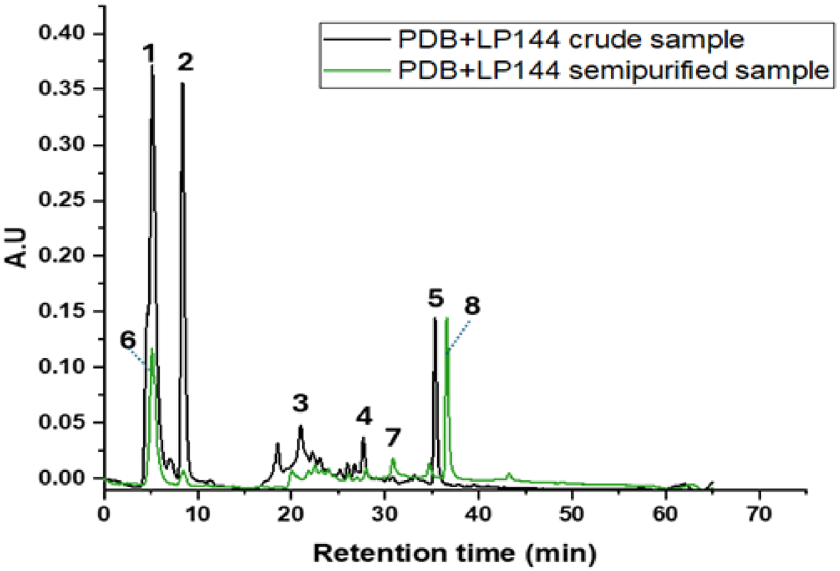

Figure S2. RP-HPLC profiles of PDB+LP144 Crude and Semi-purified extracts

Table S2. Retention times and tentative identification of compounds detected by RP-HPLC in PDB+LP144 crude and semi-purified extracts.

Sample		Retention time (min)	Compound
Crude	1	5.007	Ferulic acid 4-O-glucoside
	2	8.57	Ferulic acid 4-O-glucoside
	3	20.17	Secoisolariciresinol
	4	27.06	Ferulic acid 4-O-glucoside
	5	35.04	1-Sinapoyl-2-feruloylgentiobiose
Semi-purified	6	5.15	Ferulic acid 4-O-glucoside
	7	31.58	Spinacetin 3-O-glucosyl-(1->6)-[apiosyl(1->2)]-glucoside
	8	37.85	Quercetin 3-O-xylosyl-glucuronide
